# Unrelenting Infection of Implanted Prosthetic Hip Joint by Corynebacterium striatum

**DOI:** 10.7759/cureus.54528

**Published:** 2024-02-20

**Authors:** Supriya Sachan, Aayushi Dhawan, Vivek Jangira, Rakesh K Mahajan

**Affiliations:** 1 Microbiology, Atal Bihari Vajpayee Institute of Medical Sciences and Dr. Ram Manohar Lohla Hospital, New Delhi, IND; 2 Orthopedics, Lady Hardinge Medical College, New Delhi, IND

**Keywords:** infection prevention strategies, anti-microbial agents, multidrug resistant, virulence factors, implanted devices, biofilm, non-healing infection prosthetic joint infection, gram positive bacilli, corynebacterium striatum

## Abstract

Non-diphtherial Corynebacterial (NDC) species, while previously considered as culture contaminants, are increasingly being implicated in clinical disease and identified as causes of opportunistic infections. In cases where they grow in pure cultures, isolated from a sterile site or repeated isolations from the same patient, NDC may be labeled as clinically significant. We report here a case of non-healing infection of one of the implanted devices in a case of bilateral total hip replacement, caused by multidrug-resistant Corynebacterium striatum. Adherence to infection prevention strategies is essential for the prevention of prosthetic implant infections.

## Introduction

Corynebacterium species include organisms that are Gram-positive, catalase producers, showing club-shaped or long slender morphology. Most of the non-diphtherial Corynebacterial isolations are reported as contaminants because of their ubiquitous distribution and because some of them are also part of the commensal flora of the human skin and mucous membranes [1]. Corynebacterium species have been implicated in a variety of infections including; skin and soft-tissue, respiratory tract, postsurgical, urinary tract, cerebrospinal fluid infections, peritoneal dialysis-related infections, endocarditis and a range of different infections [2,3,4]. Corynebacterium has also been reported to cause bone and joint infection, but these are few and infrequent. Of more than one hundred species identified to date, about fifty have been associated with infections in human beings. Of these, the role of Corynebacterium striatum appears special and contentious because its pathogenic potential was reported for the first time in 1980 and is now increasingly observed as an opportunistic pathogen in hospitalized patients and those with indwelling or implanted devices not exclusively in immune-compromised situations but in the immune competent population also. While the specific virulence factors contributing to these infections are not well described, adhesion and biofilm formation have been reported to be significantly pitching into their pathogenicity [5]. C. striatum has been noted to behave quite aggressively in the presence of prosthetic implantable devices and cause persistent unrelenting infections. Historically, identifying Corynebacterium species has been challenging, problematic, and contingent on laboratories' capacity to identify these isolates but, since these non-diphtheric Corynebacteria are being reported with increasing frequency, it becomes essential that these organisms are identified up to the species level to make prudent therapeutic decisions.

In the literature search, C. amycolatum, C. aurimucosum, C. jeikeium, and C. striatum have been reported to be the most common diphtheroid species associated with prosthetic joint infections. Studies have also reported multidrug-resistant C. striatum, which was otherwise considered as a saprophyte of skin and mucous membrane to be a cause of long-standing open wound infections [6,7]. We report here a case of non-healing infection of one of the implanted devices in a case of bilateral total hip replacement, caused by multidrug-resistant Corynebacterium striatum in an apparently immune-competent adult male.

## Case presentation

A 30-year-old adult male apparently in good health presented to the orthopedic department of our institution with complaints of pain around both hip joints for about the last seven to eight years. There was a history of progressive increase in pain and associated difficulty in movement of the hip joints. Analgesics which during the initial stages of the disease provided some relief had become non-responsive. No history of trauma or fever could be elicited in the patient.

**Figure 1:**
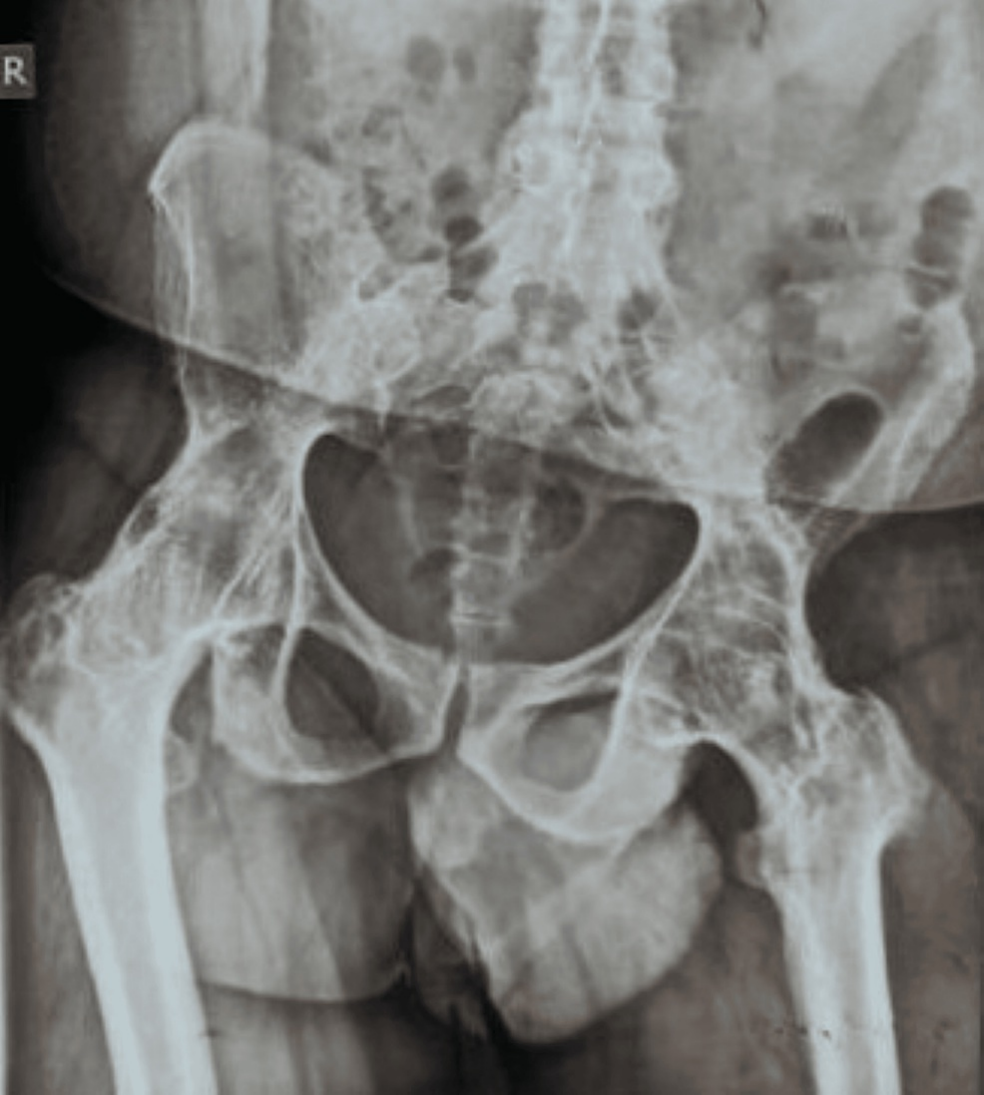
X-ray of the pelvis with both hip joints (A/P view)

**Figure 2:**
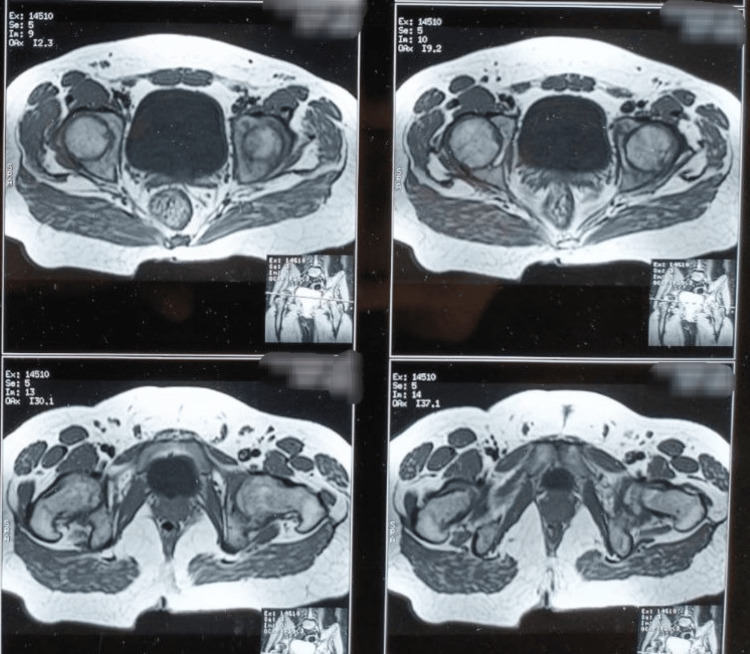
MRI of bilateral hip joints

**Figure 3:**
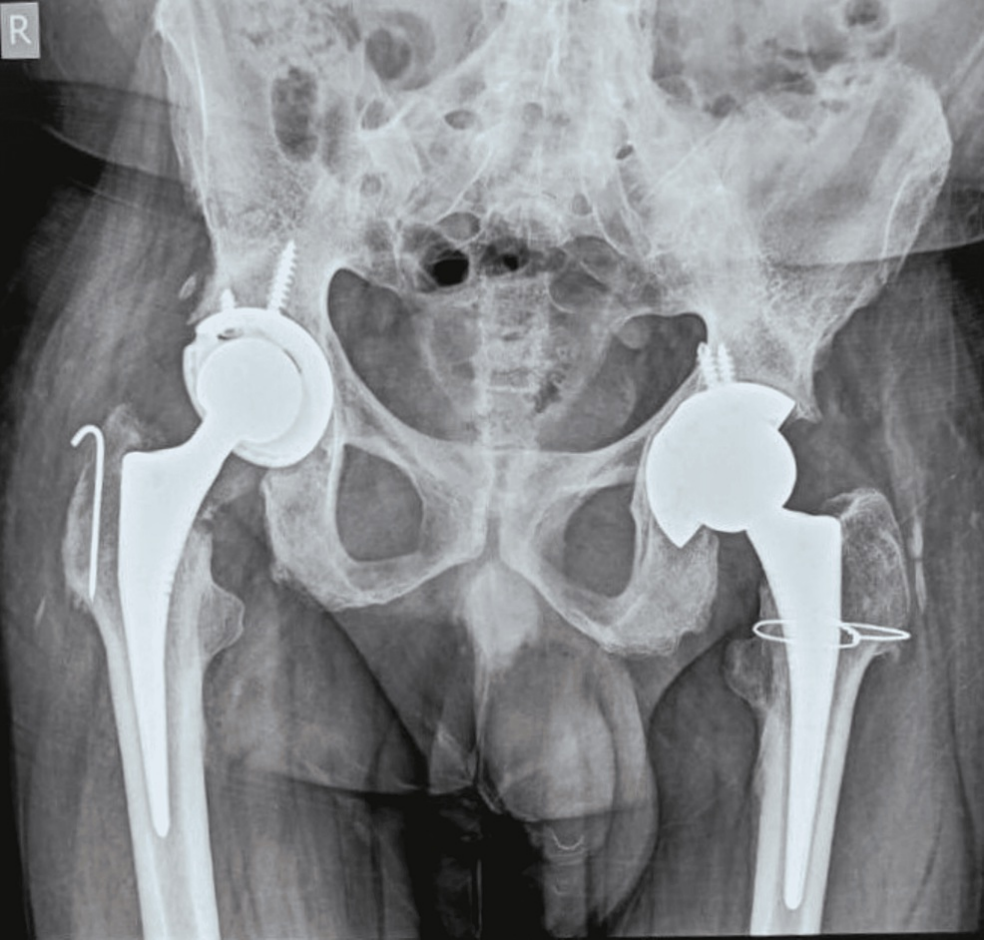
Post-operative X-ray of the pelvis with both hip joints (A/P view)

On examination, both hip joints were tender and slightly swollen, and the range of movement was significantly restricted. The X-ray of the hip joints showed features of articular osteitis of the sacroiliac joints and osteonecrosis of a subchondral portion of femoral heads involving less than one-fourth portion of the epiphysis (Figure 1). The MRI of bilateral hip and sacroiliac joints revealed sub-chondral sclerosis and narrowing of joint space, strongly indicative of ankylosing etiology (Figure 2). His blood counts were within the reference range, ruling out any infectious pathology. Serology for rheumatoid factor and anti-cyclic citrullinated peptide (anti-CCP) was also non-reactive. The only and most significant finding was his positive status for HLA B-27 antigen. He was diagnosed with a case of ankylosing spondylitis with bilateral fused hips and planned for bilateral total hip replacement. He was operated for both hips simultaneously in one go (Figure 3). He was given inj. ceftriaxone TDS, inj. amikacin BD and inj. tramadol SOS in the postoperative period. The recovery was uneventful, and post joint replacement, the patient was relieved of pain and reported significant improvement in mobility of hip joints. The patient was discharged with instructions for exercises compatible with hip replacement surgery but to avoid movements that may increase the risk of dislocation. He was asked to report to the orthopedics department in case of any worsening of pain in the hip or groin as well as issues of any deterioration of mobility of the joints.

Two months after the surgery, the patient developed a small swelling at the incision site on the right side. The swelling slowly increased in size and burst open, releasing purulent discharge. On examination, a sinus was found to be connecting the hip joint with the skin wound. A pus sample ensuring all aseptic precautions was collected and referred to the department of microbiology for culture and sensitivity. He was asked to come back after 15 days with instructions of a negative suction drain and tab cotrimoxazole.

**Figure 4:**
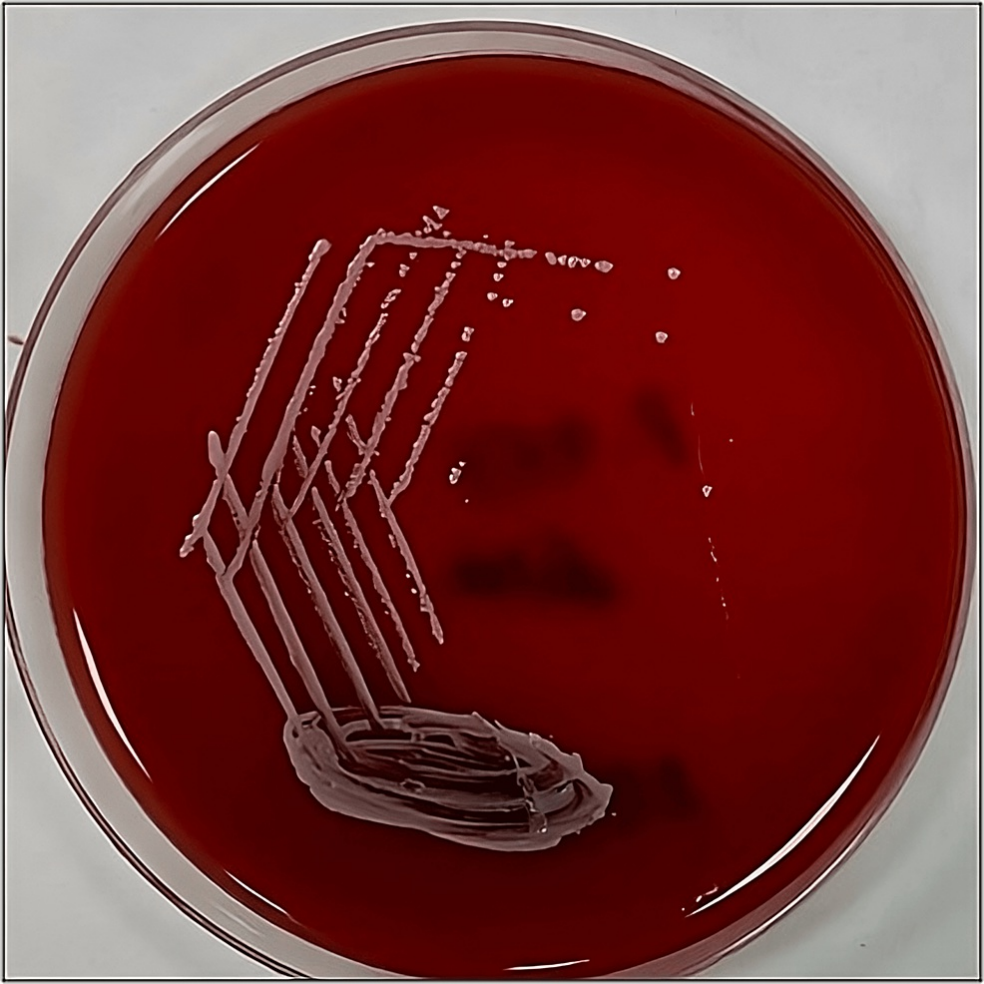
Creamy non-hemolytic colonies on 5% sheep blood agar (24 hr incubation at 37^0^C)

**Figure 5:**
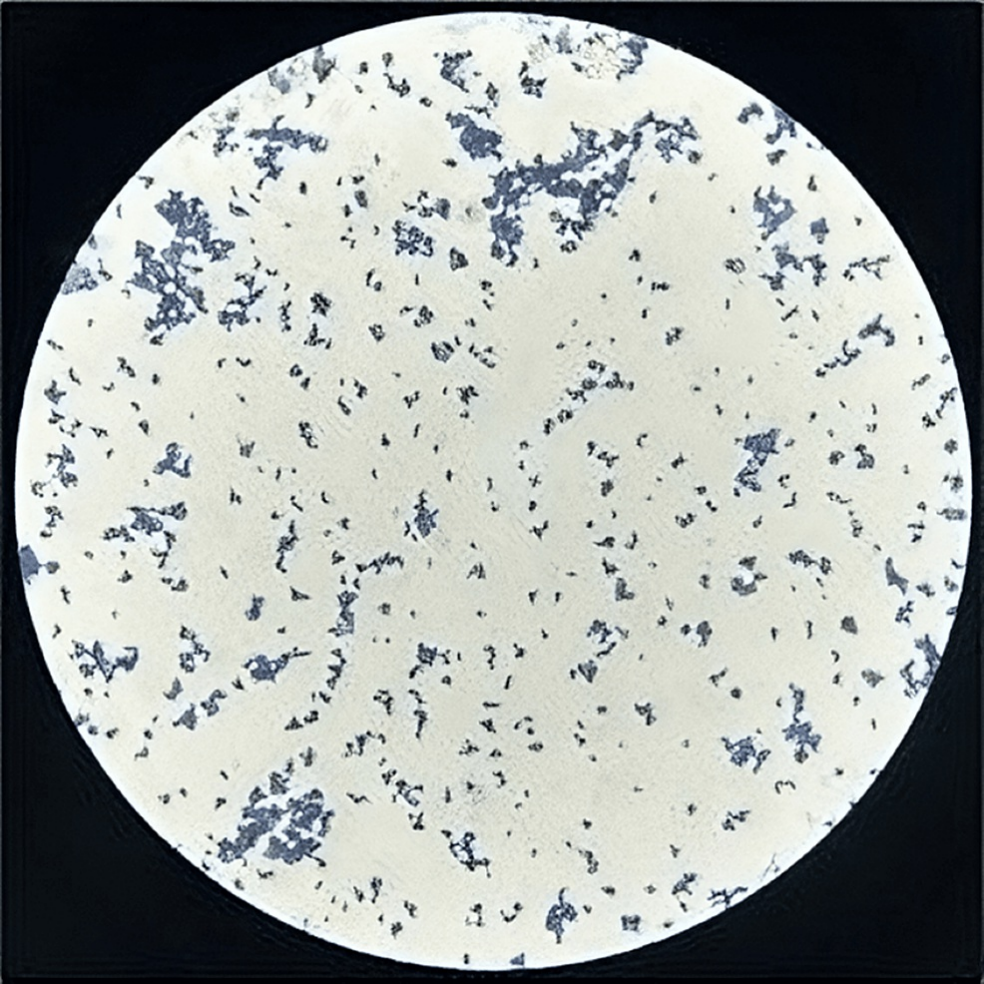
Gram-positive bacilli in palisade arrangement on gram staining

The pus sample received was subjected to Gram stain and culture onto blood agar, MacConkey agar, and Chocolate agar. Gram stain showed pus cells and a few long slender gram-positive bacilli. The culture plates were incubated at 37°C overnight. There was growth in all the three media. Colonies on blood agar were about 2 mm in size and non-hemolytic, with few small non-lactose fermenting colonies on MacConkey agar (Figure 4). Gram staining from culture plates also revealed Gram-positive bacilli arranged in a palisade arrangement, morphologically suggesting some non-diphtheric Corynebacteria (Figure 5). The culture was reported as the growth of diphtheroids, possibly a contaminant, and another sample was requested. The repeat sample also grew the non-diphtheric Corynebacteria and an effort was attempted to identify the species of the diphtheroid bacteria. The standard biochemical tests like catalase, oxidase, motility, oxidation-fermentation (OF) test, nitrate reduction, sugar fermentation (glucose, sucrose, maltose, xylose, mannitol), urease and esculin hydrolysis tests flagged some diphtheroid species only. Vitek-2 ID system gave the possibility of two to three species. Confirmation of the species Corynebacterium striatum was established with Bruker MALDI-TOF Biotyper. Antibiotic susceptibility test was performed by Kirby-Bauer disk diffusion method. Antibiotics tested include ampicillin (10mcg/disc), chloramphenicol (30mcg/disc), clindamycin (2mcg/disc), ciprofloxacin (5mcg/disc), erythromycin (10mcg/disc), gentamycin (10mcg/disc), imipenem (10mcg/disc), linezolid (30mcg/disc), penicillin (10mcg/disc), tetracycline (30mcg/disc) and vancomycin (30mcg/disc). Since the Clinical and Laboratory Standards Institute (CLSI) guidelines for the disc diffusion method for diphtheroid are lacking, we followed the method used by Reddy BS et al., [8] which had adopted the British Society for Antimicrobial Chemotherapy (BSAC) guidelines for ciprofloxacin, penicillin, and vancomycin [9] while for the other antibiotics, CLSI 2022 guidelines for Staphylococcus aureus were followed.

Three more samples collected by the treating orthopedics were forwarded for microbiological examination over the next two to three weeks and all three samples grew Corynebacterium striatum with similar antimicrobial sensitivity profile. The patient was started on oral linezolid and local infiltration and injection of vancomycin through the sinus tract for two weeks. The patient responded well to this modality, the discharge almost disappeared and there was a significant improvement in pain. The patient was asked to continue linezolid for another week and was called for follow-up after one month.

In the follow-up, it was observed that discharge and pain had not only reappeared but the quantity of discharge had increased and the patient was finding it difficult to move the joint also. It appeared to be a case of aggressive bio-film forming Corynebacterium striatum because the sample collected this time also grew this organism in pure culture. The clinical diagnosis of biofilms continues to be a challenge because there is no standardized protocol to diagnose such eventualities. According to the guidelines issued by the European Society for Clinical Microbiology and Infectious Diseases; a history of predisposition to infections such as the use of medical devices or implanted devices, clinical signs of infection persisting for more than seven days, failure of anti-microbial therapy, etc. are strong surrogate markers of infections associated with biofilm former rather than caused by an organism in planktonic state. The treating ortho team was of the opinion that the only therapeutic option available to them was to remove the implant and to plan further strategy after subjecting the area to an antibiotic spacer protocol.

## Discussion

Non-diphtherial Corynebacterial (NDC) species, while previously considered as culture contaminants, are increasingly being implicated in clinical disease and identified as causes of opportunistic infections. Long-term hospitalization, immunocompromised status, or the presence of indwelling or implanted devices are all recognized risk factors for NDC infections. It is contemplated that diagnosing infections caused by NDC bacteria is highly dependent on the capacity of the microbiology laboratories to identify these organisms. A large number of species in the NDC group coupled with a lack of well-established and standardized techniques are significant challenges in the identification of non-diphtheric Corynebacteria. It has also been suggested that molecular and chemotaxonomic analyses may be necessary to settle the species identity of organisms that have similar phenotypic characteristics.

Since an expanding trend has been observed in isolations of these organisms from immunocompromised as well as otherwise healthy hospitalized patients, their commensal status requires to be debated. In cases where they grow in pure cultures, isolated from a sterile site, or repeated isolations from the same patient, NDCs may be labeled as clinically significant. It appears essential to establish the identity of the species isolated because there does not appear to be a commonality in the expression and employment of virulence markers including sensitivity to various anti-microbial agents. This information would be extremely vital to the clinicians in planning therapeutic strategies.

Corynebacterium striatum, isolated repeatedly and consistently from this patient, in most probabilities, appears to be the culprit responsible for causing this deep-seated surgical site infection of the prosthetic hip joint. It is speculated that the organism gained entry into the joint space at the time of surgery itself because this could have been one of the predominant constituents of the skin flora around the surgical site and would have resisted the antiseptic scrubbing protocol. Lack of aggressive virulence potential may have permitted the advantage of low-level survival to the organism and, to escape the detrimental potential of anti-microbial agents could have resorted to a powerful strategy of bio-film formation. Biofilm formation on abiotic and prosthetic devices is one of the scariest situations for treating clinicians because the futility of the antimicrobial agents and the helplessness of the medical science at the hands of these micro-sized living organisms can't be more pronounced. C. striatum was sensitive to both vancomycin and linezolid besides some other drugs in in-vitro but these incredible bullets with wonderful activity against Gram-positive organisms proved completely lacking to dislodge this organism.

Adherence to infection prevention strategies is essential and integral to the safe delivery of health care and the prevention of surgical site infections. The application of standard precautions is a fundamental practice that is mandatory at all times of patient care and management. Standard precautions include but are not limited to; hand hygiene, a clean environment commensurate with the type and criticality of health care, use of personal protective equipment (PPE), strict compliance to cough etiquette, management of biomedical waste, etc. [10]. Any non-compliance to infection prevention and control (IPC) protocols may result in adverse outcomes with significant unacceptable morbidity.

Infection prevention strategies are a package of inter-connected procedures and some gaps in this whole process can't be ruled out even in the best of hands and healthcare facilities. So, it becomes essential that we explore other options including recent technologies to fight the problem of surgical site infections (SSIs). Implant-associated infections represent some of the most serious and dangerous complications. Of the various strategies, designing metal materials with antibacterial activity or alloying of existing materials like titanium, cobalt, bio-degradable materials, etc. may provide significant protection against the invading unforeseen eventualities [11].

## Conclusions

The isolation and identification of non-diphtherial Corynebacteria remain a challenge for microbiology laboratories and there is an urgent need for the labs to upgrade their diagnostic systems to manage such organisms so that initial concerns could be flagged to the treating clinicians at the earliest for planning and instituting appropriate therapeutics. Needless to underscore the sanctity of compliance to infection prevention protocols and disposal of bio-medical waste. Clinicians need to be sensitized to these emerging issues of infections by low-virulence organisms which may be difficult to pick up in the early stages. Prosthetic implant surgeries will have to explore the use of technologies like the use of materials with an antibacterial spectrum.

## References

[1] Smith JW, Chalupa P, Shabaz Hasan M (2006). Infectious arthritis: clinical features, laboratory findings and treatment. Clin Microbiol Infect.

[2] Chandran R, Puthukkichal DR, Suman E, Mangalore SK (2016). Diphtheroids-important nosocomial pathogens. J Clin Diagn Res.

[3] Cazanave C, Greenwood-Quaintance KE, Hanssen AD, Patel R (2012). Corynebacterium prosthetic joint infection. J Clin Microbiol.

[4] Hong HL, Koh HI, Lee AJ (2016). Native valve endocarditis due to Corynebacterium striatum confirmed by 16s ribosomal RNA sequencing: a case report and literature review. Infect Chemother.

[5] Høiby N, Bjarnsholt T, Moser C (2015). ESCMID guideline for the diagnosis and treatment of biofilm infections 2014. Clin Microbiol Infect.

[6] Biswal I, Mohapatra S, Deb M, Dawar R, Gaind R (2014). Corynebacterium striatum: an emerging nosocomial pathogen in a case of laryngeal carcinoma. Indian J Med Microbiol.

[7] Westblade LF, Shams F, Duong S (2014). Septic arthritis of a native knee joint due to Corynebacterium striatum. J Clin Microbiol.

[8] Reddy BS, Chaudhury A, Kalawat U, Jayaprada R, Reddy G, Ramana BV (2012). Isolation, speciation, and antibiogram of clinically relevant non-diphtherial Corynebacteria (diphtheroids). Indian J Med Microbiol.

[9] Andrews JM (2008). BSAC standardized disc susceptibility testing method (version 7). J Antimicrob Chemother.

[10] (2015). Mandell, Douglas, and Bennett's Principles and Practice of Infectious Diseases. Mandell, Douglas, and Bennet Principles and Practice of Infectious Diseases.

[11] Zhang E, Zhao X, Hu J, Wang R, Fu S, Qin G (2021). Antibacterial metals and alloys for potential biomedical implants. Bioact Mater.

